# Differences in Contractile Characteristics Among Various Muscle Groups in Youth Elite Female Team Handball Players Compared to a Control Group

**DOI:** 10.3390/sports13020027

**Published:** 2025-01-21

**Authors:** Milan Petronijević, Katarina Ohnjec, Milivoj Dopsaj

**Affiliations:** 1 Faculty of Sport and Physical Education, University of Belgrade, 11221 Belgrade, Serbia; milan.petronijevic@fsfv.bg.ac.rs (M.P.); milivoj.dopsaj@fsfv.bg.ac.rs (M.D.); 2 Faculty of Kinesiology, University of Zagreb, 10000 Zagreb, Croatia

**Keywords:** contractile characteristics, body partialization, team sport, youth athletes

## Abstract

Muscular strength and explosiveness are generally known as factors that affect physical performance. Physical ability modeling has a profound connection with long-term athlete development and talent identification. The purpose of the current study was to investigate differences in contractile characteristics (maximal isometric force and maximal rate of force development) among various muscle groups in youth elite female team handball players (*n* = 35, 16.6 ± 1.1 years) compared to a control group (*n* = 28, 16.7 ± 1.1 years). The following tests were performed: isometric non-dominant hand grip, isometric dominant hand grip, isometric deadlift, isometric standing leg extension, and isometric bilateral ankle extension. For each subject, the maximal isometric force and maximal rate of force development were derived from the isometric strength tests. The research analyzed a total of twenty-four variables, presented in both absolute and relative values. Statistical analysis revealed significant differences among all pairs of variables in the absolute values of maximal isometric force (Λ = 0.531, F = 10.07, *p* = 0.000) and maximal rate of force development (Λ = 0.692, F = 5.08, *p* = 0.001) between the two groups. The most significant difference was found in the grip of the dominant hand, where the impact of the difference was 43.6% and 37.0% for the absolute values of force and explosiveness. Conversely, no differences were observed between the pairs of variables representing the relative values across the two subject groups, except for the dominant hand grip relative force (*p* = 0.006). The results provide information about the contractile potentials of important muscle groups in the game of handball, which could help in adapting strength training according to the specifics of the strength and explosivity development of young female handball players.

## 1. Introduction

Handball is one of the leading sports worldwide, thriving in countries of different sizes and economies [1]. This dynamic and physically demanding team sport is characterized by intense physical contact, so to optimize a player’s performance, they require well-honed motor skills, which include speed, strength, power, and endurance [2,3]. Since handball is a game characterized by significant, yet mostly permissible contact, it fosters the balanced development of an athlete’s motor functions [4]. To gain a deeper understanding of an athlete’s characteristics, the impact of training, and their career development, it is essential to perform some control procedures as well as to monitor the athlete’s physical abilities systematically [1,5,6]. The modeling of physical abilities is a vital process in managing the development of young athletes, which provides crucial and objective data that help in talent identification and in the development of training programs [7,8]. Moreover, weak muscle strength and impaired neuromuscular control have been suggested as risk factors [9,10] and could therefore, if detected early enough, help in injury prevention.

Muscular strength is a crucial determinant of sports performance, with high levels of speed and muscular strength required for the optimal execution of jumps, movements, throws, pushes, and directional changes in both offensive and defensive phases of the game of handball [11,12]. Numerous studies have emphasized the importance of optimal intermuscular coordination, strength, and power as key factors in achieving peak performance in handball, for both male and female players [2,13,14]. Most of the decisive movements in a handball match, such as accelerations, sprinting, tackles, jumping, and throwing, demand a high level of explosiveness and strength [15]. The ability to perform rapid and forceful muscle contractions represents a physiological advantage in team handball gameplay [16]. Much research has been conducted to study different training operations applied during handball training which focus on improving isokinetic, isometric, and maximal strength, muscle power, and ball throwing velocity [10,15,17,18]. Female handball players of different ages, competition ranks, experience levels, and playing positions have been studied in relation to their muscular strength and explosiveness. The players have often been analyzed on the basis of their physical and physiological characteristics and performance profiles [4,19,20,21], as well as through the performing of modeling procedures which involved various training interventions aimed at improving their abilities [10,15,16,18,22]. For young female handball players (U-16), different programs of strength training [23,24] improved physical performance (strength, sprint, change of direction, jump, and repeated change of direction), which is essential in the performance of the technical and tactical elements of the game of handball.

Hand grip strength is a determinant for the catching, passing, and throwing of the ball in handball [20]; therefore, the hand grip test is an integral part of the battery of tests for handball players. Among the different fitness components, cardiorespiratory fitness and muscular strength have shown the strongest and most consistent health-related connection and are consequently considered the main health-related fitness components in children and adolescents [25]. One of the tests that is recommended as indicative of overall muscle strength is the grip strength test. Normalized hand grip strength in adolescents is associated with both cardiometabolic health maintenance and health improvements [26].

The major role of lower-limb strength and power has also been highlighted in many studies demonstrating a significant relationship between high intensity actions and lower-limb strength/power [17]. Mechanical muscle function in the lower limbs can be evaluated by means of isometric dynamometry, which is typically used to measure isolated single-joint muscle strength (peak torque) and the rate of torque development (RTD), where RTD (Δtorque/Δtime) can be derived from the torque–time curve obtained during maximal voluntary isometric contraction (MVIC) [27,28,29]. Research on top female handball players [16] with results demonstrating their maximal lower-limb strength emphasizes the importance of the obtained facts in indicating a predisposition to ACL injuries, as well as the practical importance of these variables for optimizing athlete performance.

Evidently, there is a lack of sufficient scientific data regarding the topological structure of the maximal isometric force (F_max_) and maximal rate of force development (RFD_max_) not only in team handball but also across team sports in general. Furthermore, a review of handball players’ isokinetic, isometric, and maximal strength and muscle power indicates that young handball players are less represented as research subjects [17].

This study aims to investigate the differences in the contractile characteristics (maximal isometric force and maximal rate of force development) of various muscle groups in youth elite female team handball players and high school female students with no sports training experience.

## 2. Materials and Methods

### 2.1. Participants

This study’s sample of subjects comprised 53 young females, divided into two groups. The first subgroup included 35 female team handball players from the Serbian Youth National Team, selected during a preparatory training camp (age of 16.6 ± 1.1 years, body height of 173.2 ± 5.9 cm, body mass of 69.7 ± 7.9 kg, body mass index of 23.21 ± 1.96 kg/m^2^, and training experience of 7.4 ± 2.8 years). The second group consisted of 28 randomly selected high school female students with no sports training experience (age of 16.7 ± 1.1 years, body height of 165.2 ± 6.1 cm, body mass of 54.5 ± 6.1 kg, and body mass index of 19.94 ± 1.78 kg/m^2^). The research was conducted as a cross-sectional study, with all measurements performed in a laboratory setting. We utilized a dynamometric method to assess muscle contractile characteristics, employing a highly sensitive tensiometric probe and standardized testing procedures [5,6,30,31]. This study was conducted in accordance with the requirements of the Helsinki Declaration and the recommendations guiding physicians in biomedical research involving human subjects [32]. The research was approved by the Ethics Commission of the Faculty of Sport and Physical Education, University of Belgrade (Number 484-2).

### 2.2. Testing Procedure

All tests were conducted at the conclusion of the main preparatory mesocycle for the 2022/23 competition season of the National Junior Team at the Methodological Research Testing Laboratory of the Faculty of Sports and Physical Education, University of Belgrade (MIL). The testing protocol comprised a single experimental session, held in the morning between 9:00 and 12:00 AM, aligning with the participants’ regular macrocycle training schedule. Subjects were instructed to refrain from strenuous exercise for at least 48 h prior to testing and to avoid eating for a minimum of 1.5 h before the session. All participants were familiar with the testing methodology employed.

The testing battery consisted of five individual tests—isometric non-dominant hand grip (HG_Nd), isometric dominant hand grip (HG_Do), isometric deadlift (DL), isometric standing leg extension (LE), and isometric bilateral ankle extension (AE)—where the validity and reliability of the applied isometric method in relation to the tests used were in the range of ICC = 0.89–0.99 for F_max_ and ICC = 0.76–0.98 for RFD_max_ [6,33,34]. Also, the summation of all individual tests resulted in a new variable that was a general indicator of overall body strength or explosiveness, which was also expressed in absolute (SUM_F_max_ and SUM_RFD_max_) and relative values (SUM_F_rel_ and SUM_RFD_rel_). All tests were conducted following established procedures [6,30,31]. For each isometric strength test, two contractile characteristics were analyzed: maximal isometric force (F_max_) and maximal isometric rate of force development (RFD_max_).

Measurements were taken using a fixed force transducer (Hottinger, Type S9, Darmstadt, Germany; tensile/compressive sensitivity: 2 mV/N) and specially designed setups. A dedicated software–hardware system (Isometrics Lite, ver. 3.1.1) was employed for data collection and analysis. The force–time signal was sampled at 1000 Hz and low-pass filtered (10 Hz) using a fourth-order Butterworth filter [35]. The onset of contraction was defined as the moment when the first derivative of the force–time curve exceeded the baseline by 3% of its maximum value, with gravity corrections applied by subtracting the baseline force in the resting state from all active-phase force recordings.

The testing procedure followed this protocol: Each session began with a 5 min general warm-up, which included 3 min of jogging followed by dynamic stretching for both the upper and lower body. This was followed by a specific 3 min warm-up tailored to each test, incorporating dynamic exercises targeting the muscle group to be assessed. Each subject completed two submaximal warm-up attempts to refamiliarize themselves with the testing protocol. After a 5 min passive rest, subjects performed three maximal trials, with the best result recorded for further analysis. The order of tests was randomized for each group of subjects, with a 10 min rest period between tests and a 2 min rest between trials.

The research analyzed a total of twenty-four (24) variables, presented in both absolute and relative values. The absolute values for all individual tests were expressed in Newtons (N) for maximal isometric force (F_max_) and in Newtons per second (N/s) for maximal isometric rate of force development (RFD_max_). The relative values (F_rel_ and RFD_rel_) were calculated using a previously defined and published method for partializing isometric strength and explosiveness [33]. For all independent tests, the relative values of maximal isometric force were expressed in Newtons per body mass in kg corrected by allometric partialization (N/BM^0.667^), and those for the maximal isometric rate of force development were expressed in Newtons per second per skeletal muscle mass index (N/s/SMMI).

### 2.3. Statistical Analysis

Descriptive statistics including the Mean and Standard Deviation (SD) were calculated for all explored variables. The normality of the distribution was assessed using the Kolmogorov–Smirnov non-parametric test (KSZ). To establish differences between the absolute and relative values of maximal isometric force (F_max_ and F_rel_) and absolute and relative values of maximal rate of force development (RFD_max_ and RFD_rel_), a multivariate analysis of variance (MANOVA) was employed in order to confirm the existence of general differences in the investigated multivariate space (multiple variables) of subsamples. A univariate analysis of variance (ANOVA) was employed in order to determine the variance and difference in variance between pairs of the variables of the examined subsamples, and *t*-tests with Bonferroni corrections were employed for paired sample differences [34]. Sample size and statistical power were calculated using the specialized software G*Power Version 3.1.9.4 (Copyright © 1992–2019) and amounted to a minimum of 48 respondents for an actual power of 0.958. All statistical analyses were conducted using IBM SPSS Statistics version 23 (Release. 23.0.0.0., IBM Corp., Armonk, NY, USA, 2015), with a significance level set at *p* < 0.05.

## 3. Results

Table 1 shows the results of descriptive statistics and the differences in the absolute values of the maximum isometric strength variables in relation to the examined groups (handball players and the control group). The existence of a general statistically significant difference (multivariate test—MANOVA) was established at the level of Wilks’ lambda value = 0.531; F relation = 10.07; and *p* = 0.000. The variance in the investigated variables responsible for the determined difference was at a level of 46.9% (Part. Eta^2^ = 0.469), while the observed power was 1.000 (100%). Between all pairs of variables, a statistically significant difference was found between *p* = 0.001 and 0.000 (Table 1).

Table 2 shows the results for descriptive statistics and the differences in the absolute values of the maximum isometric explosive strength variables in relation to the examined groups. The existence of a general statistically significant difference (MANOVA) was established at the level of Wilks’ lambda value = 0.692; F relation = 5.08; and *p* = 0.001. The variance in the investigated variables responsible for the determined difference was at a level of 30.8% (Part. Eta^2^ = 0.308), while the observed power was 0.967 (96.7%). Between all pairs of variables, a statistically significant difference was found between *p* = 0.004 and 0.000 (Table 2).

Table 3 shows the results for descriptive statistics and the differences in the relative values of the maximum isometric strength variables in relation to the examined groups. The existence of a general statistically significant difference (MANOVA) was established at the level of Wilks’ lambda value = 0.784; F relation = 2.58; and *p* = 0.028. The variance in the investigated variables responsible for the determined difference was at a level of 21.6% (Part. Eta^2^ = 0.216), while the observed power was 0.807 (80.7%). In the case of the relative value of the maximum isometric strength, a statistically significant difference was found in only one pair of variables (HG_F_rel__Do, *p* = 0.006), while there were no observed differences in all the other pairs of variables (Table 3).

Table 4 shows the results for descriptive statistics and the differences in the relative values of the maximum isometric explosive strength variables in relation to the examined groups. A general statistically significant difference do not existed only in the case of the relative values of maximum isometric explosiveness (MANOVA: Wilks’ lambda value = 0.929; F relation = 0.87; *p* = 0.505; Part. Eta^2^ = 0.071; observed power = 0.290).

Also, through the application of the Kolmogorov–Smirnov non-parametric test (KSZ) for all variables considering the examined groups, it was determined that all variables were normally distributed.

**Figure 1:**
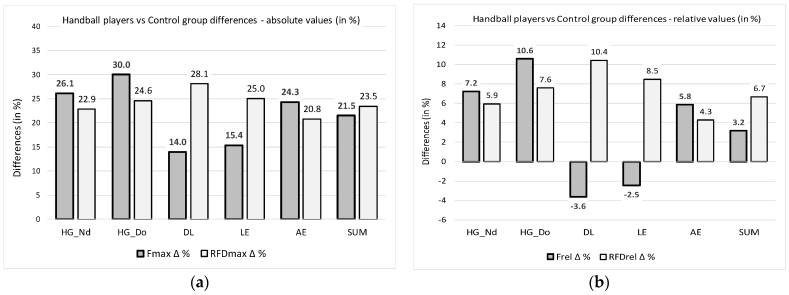
(**a**) Percentage differences in the examined muscle groups (variables) between the two groups in relation to the F_max_ and RFD_max_ absolute values. (**b**) Percentage differences in the examined muscle groups (variables) between the two groups in relation to the relativized values of F_max_ and RFD_max_.

Figure 1a displays the percentage differences in the examined muscle groups (variables) between the two groups, based on the absolute values of F_max_ and RFD_max_. The results show that in the case of the absolute values of F_max_ and RFD_max_ between the handball players and the control group (Table 1 and Table 2, Figure 1a), there are statistically significant differences in all pairs of variables as well as from DL_F_max_—*p* = 0.001; Part. Eta^2^ = 0.156; 14.0%—to HG_F_max__Do—*p* = 0.000; Part. Eta^2^ = 0.436; 30.0%—and from LE_RFD_max_—*p* = 0.004; Part. Eta^2^ = 0.125; 25.0%; to HG_RFD_max__Do—*p* = 0.000; Part. Eta^2^ = 0.270; 24.6%—respectively.

Figure 1b displays percentage differences in the examined muscle groups (variables) between the two groups, based on the relativized values of F_max_ and RFD_max_. The results indicate that when considering the relative values of F_max_ and RFD_max_, there are notable differences between the handball players and the control group (Table 3, Figure 1b). Specifically, for the relative values of maximum isometric strength, a significant difference is observed in only one variable: HG_F_rel__Do, with *p* = 0.006 and Part. Eta^2^ = 0.119, corresponding to a 10.6% difference. No significant differences were found in any of the other muscle groups. Regarding the relative values of explosiveness, no statistically significant differences were identified for any variables (Table 4, Figure 1b).

## 4. Discussion

The main topic of this research is the contractile characteristics (maximal isometric force and maximal rate of force development) of various muscle groups in youth elite female team handball players and girls of the same age without any experience in sports. The main findings of this study indicate significant differences among all pairs of variables in the absolute values of maximal isometric force and maximal rate of force development between the two groups, whereas relative values show no such correlation.

Young female handball players’ maximal isometric hand grip results obtained in this study (Table 1) are slightly lower than in previous studies that observed similar groups of subjects, with authors reporting F_max_ results for U16 female handball players in the range of 354.2 ± 50.9 N for the dominant hand, and 323.8 ±35.5 N for the non-dominant hand [31]. In a sample of 20-year-old female handball players, the corresponding values for hand grip were higher at 430 N ± 63 and 405 N ± 53 [31]. Handball causes an asymmetrical increase in the hypertrophy of body musculature in professional athletes, indicating a greater muscle mass of the right upper limb and significantly higher grip strength. Therefore, it is important to aim for individualized symmetrization during sports practice and to consistently monitor the asymmetries occurring in different body parts, which could contribute to an improvement in players’ sports results and to the minimization of injury risk [36].

Research on the maximal isometric force and maximal rate of force for body segments (arms, trunk, and legs) in a population of younger female handball players, which applied different procedures (testing, instruments) [15,22], shows consistently comparable results to a study of young female volleyball players [6]. Compared to young U17 volleyball players, the results are slightly higher in isometric hand grip (290.1 ± 47.6 for the dominant hand, 273.49 ± 46.4 for the non-dominant hand), while similar results in the maximal force and rate of force development for lumbar extensors and ankle extensors have been recorded [6].

The same measurement method and battery of tests were used on a sample of 75 healthy and physically active female students aged 23.0 years [33]. It was determined that they had an average value of general strength at a level of 5726 N, and general explosiveness at a level of 32,449 N/s. The relative values of the investigated variables calculated by the same method were at the levels of 714.5 N/kg^0.667^ and 3460.3 N/s/SMMI for the relative values of maximum force and maximum explosiveness, respectively [33]. The handball players from the sample were, on a general level, stronger (the SUM_F_max_ absolute value was higher at 321 N, i.e., relatively higher by 5.61%) than the general population of adult physically active young women, but the control group, on a general level, was weaker, with an absolute value of 749 N and a relative value of 13.08%. Similar results were found for the general level of maximum explosiveness (SUM_RFD_max_), where handball players were more explosive than young women (at 1271 N/s and 3.92%), but girls from the control group were less explosive than young women, at 5141 N/S and 15.84%.

Based on these comparisons, it can be concluded that handball training has a positive effect on increasing the general level of muscle strength and explosiveness by 3.92 and 5.61%, respectively, in girls aged 16–17 years compared to adult physically active young women aged 23.0 years.

Experts have been extremely interested in research on the anthropo-morphological characteristics and body composition of children [37] and adolescents [38,39] and the influence these characteristics have on the children and adolescents’ performance in motor tests assessing strength, body coordination, and aerobic fitness. The conclusions predominantly suggest a reduced overall physical preparedness of subjects in comparison to previous generations [40], and they stress the necessity of physical activity to improve general health and physical fitness and to maintain optimal levels of fat mass, etc. The isometric hand grip results in this study obtained for randomly selected high school female students are similar to the results of other studies of the population of high school adolescents who have no experience in sports activities.

The tested sample of young female handball players demonstrated statistically significant advantages (Table 1 and Table 2) in strength and explosiveness in terms of the absolute values of contractile characteristics measured in the subjects (Table 3 and Table 4). On the contrary, no differences were observed between the pairs of variables representing the relative values of maximal isometric force and maximal rate of force development across the two subject groups. A muscle’s ability to achieve maximum isometric strength and a muscle’s ability to exhibit maximum isometric explosiveness in different muscle groups of all three body segments (arms, trunk, and legs) are significantly higher in young female handball players than in the population of high school girls. However, these results appear to be due to the physical superiority of the handball players (taller and with higher BMI) rather than due to the genuinely higher contractile potential of the tested muscle groups. Targeted handball training aimed at the overall young female athletes’ development, which can be observed through improvements in all their abilities, as well as technical and tactical skills applied in decision-making during handball matches [41], induces a positive transition in relation to maximum strength and explosive strength. The monitoring and control of an athlete’s long-term development and talent identification [42], as deterministic systems, require an understanding of the developmental trends in all features of the athlete’s physiology and psychology which are crucial for her performance [5]. Improvement in the performance of young female handball players in different strength and power tests can be achieved by applying different types of training, such as strength and plyometric training [22,43] and explosive resistance training [15], which suggests that these should be implemented in systematic training work with young handball players.

It is necessary that all kinesiology experts in different educational environments promote improvements in the components of physical fitness in adolescents. In this context, international organizations, such as the American College of Sports and Medicine and the World Health Organization, recommend the promotion of aerobic and muscle-strengthening activities (of moderate-to-vigorous and/or high intensity) as part of the physical activity guidelines for children and adolescents [44].

This study emphasizes the importance of monitoring the isometric strength and speed of force development (explosiveness) in young female handball players as well as in the population of female high school adolescents. A notable limitation of this study is the lack of comparable research findings for analysis. Future research should focus on examining senior female handball players and expanding the scope to male handball players and athletes from other team sports.

## 5. Conclusions

The selected tests and procedures offer important information about the absolute and relativized values of the F_max_ and RFD_max_ results of important muscle groups in the game of handball. Modeling young female handball players’ contractile potentials provides important information for creating strength training procedures and monitoring the effects of training in long-term athlete development. Coaches might find these values useful in detecting young female handball athletes who stand out as strong regarding their age, and clinicians could identify deficient athletes as being at risk of possible injury.

## Figures and Tables

**Table 1:** Descriptive statistics and differences between absolute values of maximal isometric strength variables.

	Handball Players	Control Group	Univariate Tests			
Variables	Mean ± SD	Mean ± SD	F Relation	*p*	Part. Eta^2^	Obs. Power
HG_F_max__Nd	304 ± 41	241 ± 40	38.14	0.000	0.385	1.000
HG_F_max__Do	338 ± 47	260 ± 42	47.13	0.000	0.436	1.000
DL_F_max_	962 ± 139	844 ± 136	11.30	0.001	0.156	0.911
LE_F_max_	938 ± 111	813 ± 169	12.57	0.001	0.171	0.937
AE_F_max_	3505 ± 535	2819 ± 459	28.96	0.000	0.322	1.000
SUM_F_max_	6047 ± 752	4977 ± 621	36.87	0.000	0.376	1.000

HG_F_max__Nd—isometric non-dominant hand grip maximal force; HG_F_max__Do—isometric dominant hand grip maximal force; DL_F_max_—isometric deadlift maximal force; LE_F_max_—isometric standing leg extension maximal force; AE_F_max_—isometric bilateral ankle extension maximal force; SUM_F_max_—sum of maximal force values among all tests.

**Table 2:** Descriptive statistics and differences between absolute values of maximal explosive isometric strength variables.

	Handball Players	Control Group	Univariate Tests			
Variables	Mean ± SD	Mean ± SD	F Relation	*p*	Part. Eta^2^	Obs. Power
HG_RFD_max__Nd	1913 ± 368	1557 ± 321	16.26	0.000	0.210	0.978
HG_RFD_max__Do	2181 ± 387	1750 ± 319	22.52	0.000	0.270	0.997
DL_RFD_max_	6976 ± 1821	5446 ± 1493	12.85	0.001	0.174	0.942
LE_RFD_max_	6860 ± 1835	5486 ± 1835	8.73	0.004	0.125	0.828
AE_RFD_max_	15790 ± 2846	13071 ± 2786	14.47	0.000	0.192	0.963
SUM_RFD_max_	33720 ± 5479	27308 ± 5239	22.14	0.000	0.266	0.996

HG_RFD_max__Nd—isometric non-dominant hand grip maximal explosive force; HG_RFD_max__Do—isometric dominant hand grip maximal explosive force; DL_RFD_max_—isometric deadlift maximal explosive force; LE_RFD_max_—isometric standing leg extension maximal explosive force; AE_RFD_max_—isometric bilateral ankle extension maximal explosive force; SUM_RFD_max_—sum of maximal explosive force values among all tests.

**Table 3:** Descriptive statistics and differences between relative values of maximal isometric strength variables.

	Handball Players	Control Group	Univariate Tests			
Variables	Mean ± SD	Mean ± SD	F Relation	*p*	Part. Eta^2^	Obs. Power
HG_F_rel__Nd	17.99 ± 2.17	16.78 ± 2.70	3.92	0.05	0.060	0.495
HG_F_rel__Do	19.99 ± 2.63	18.08 ± 2.61	8.28	0.006	0.119	0.808
DL_F_rel_	56.84 ± 7.64	58.99 ± 10.15	0.92	0.341	0.015	0.157
LE_F_rel_	55.48 ± 6.04	56.88 ± 13.08	0.32	0.574	0.005	0.086
AE_F_rel_	207.08 ± 27.77	195.65 ± 24.55	2.91	0.093	0.046	0.390
SUM_F_rel_	357.38 ± 38.24	346.39 ± 38.22	1.29	0.261	0.021	0.201

HG_Frel_Nd—isometric non-dominant hand grip relative value of maximal force; HG_F_rel__Do—isometric dominant hand grip relative value of maximal force; DL_F_rel_—isometric deadlift relative value of maximal force; LE_F_rel_—isometric standing leg extension relative value of maximal force; AE_F_rel_—isometric bilateral ankle extension relative value of maximal force; SUM_F_rel_—sum of relative values of maximal force among all tests.

**Table 4:** Descriptive statistics and differences between relative values of maximal explosive isometric strength variables.

	Handball Players	Control Group	Univariate Tests			
Variables	Mean ± SD	Mean ± SD	F Relation	*p*	Part. Eta^2^	Obs. Power
HG_RFD_rel__Nd	190.22 ± 30.35	179.56 ± 31.50	1.86	0.178	0.030	0.269
HG_RFD_rel__Do	217.32 ± 32.95	202.02 ± 30.03	3.63	0.062	0.056	0.466
DL_RFD_rel_	695.73 ± 177.52	630.02 ± 164.42	2.28	0.137	0.036	0.317
LE_RFD_rel_	684.77 ± 183.58	631.29 ± 197.17	1.24	0.271	0.020	0.194
AE_RFD_rel_	1573.38 ± 246.61	1508.90 ± 285.31	0.93	0.340	0.015	0.157
SUM_RFD_rel_	3361.43 ± 483.16	3151.78 ± 518.86	2.74	0.103	0.043	0.371

HG_RFD_rel__Nd—isometric non-dominant hand grip relative value of maximal rate of force development; HG_RFD_rel__Do—isometric dominant hand grip relative value of maximal rate of force development; DL_RFD_rel_—isometric deadlift relative value of maximal rate of force development; LE_RFD_rel_—isometric standing leg extension relative value of maximal rate of force development; AE_RFD_rel_—isometric bilateral ankle extension relative value of maximal rate of force development; SUM_RFD_rel_—sum of relative values of maximal rate of force development among all tests.

## Data Availability

Individual data are available upon request to the first author.
